# Interplay between polygenic propensity for ageing-related traits and the consumption of fruits and vegetables on future dementia diagnosis

**DOI:** 10.1186/s12888-022-03717-5

**Published:** 2022-01-30

**Authors:** Emma Ruby Francis, Dorina Cadar, Andrew Steptoe, Olesya Ajnakina

**Affiliations:** 1 Department of Behavioural Science and Health, Institute of Epidemiology and Health Care, University College London, 1-19 Torrington Place, London, WC1E 7HB UK; 2 Brighton and Sussex Medical School, Brighton, East Sussex, UK; 3 Department of Biostatistics & Health Informatics, Institute of Psychiatry, Psychology and Neuroscience, King’s College London, London, UK

**Keywords:** Alzheimer’s disease, Dementia, Diet, Fruits and vegetables, Polygenic susceptibility

## Abstract

**Background:**

Understanding how polygenic scores for ageing-related traits interact with diet in determining a future dementia including Alzheimer’s diagnosis (AD) would increase our understanding of mechanisms underlying dementia onset.

**Methods:**

Using 6784 population representative adults aged ≥50 years from the English Longitudinal Study of Ageing, we employed accelerated failure time survival model to investigate interactions between polygenic scores for AD (AD-PGS), schizophrenia (SZ-PGS) and general cognition (GC-PGS) and the baseline daily fruit and vegetable intake in association with dementia diagnosis during a 10-year follow-up. The baseline sample was obtained from waves 3–4 (2006–2009); follow-up data came from wave 5 (2010–2011) to wave 8 (2016–2017).

**Results:**

Consuming < 5 portions of fruit and vegetables a day was associated with 33–37% greater risk for dementia in the following 10 years depending on an individual polygenic propensity. One standard deviation (1-SD) increase in AD-PGS was associated with 24% higher risk of dementia and 47% higher risk for AD diagnosis. 1-SD increase in SZ-PGS was associated with an increased risk of AD diagnosis by 66%(95%CI = 1.05–2.64) in participants who consumed < 5 portions of fruit or vegetables. There was a significant additive interaction between GC-PGS and < 5 portions of the baseline daily intake of fruit and vegetables in association with AD diagnosis during the 10-year follow-up (RERI = 0.70, 95%CI = 0.09–4.82; AP = 0.36, 95%CI = 0.17–0.66).

**Conclusion:**

A diet rich in fruit and vegetables is an important factor influencing the subsequent risk of dementia in the 10 years follow-up, especially in the context of polygenetic predisposition to AD, schizophrenia, and general cognition.

**Supplementary Information:**

The online version contains supplementary material available at 10.1186/s12888-022-03717-5.

## Introduction

Dementia, of which approximately two-thirds constitute Alzheimer’s Disease (AD) cases [1], is associated with a progressive decline of brain functioning leading to a significant loss of autonomy, reduced quality of life and a shortened life expectancy [2]. Confronting the growing burden of dementia requires an identification of the mechanisms by which its risk is exacerbated or attenuated, especially if they entail modifiable risk factors. In turn, this can highlight ways to reduce disease occurrence in the general population.

Considering there is a substantial genetic contribution to dementia [3] and AD [4], one approach to measuring the genetic susceptibility to this disease has been polygenic score (PGSs) analyses [5], which demonstrated that individual differences in risk for dementia and AD diagnoses are driven by thousands of common genetic markers associated with AD [3, 6, 7]. PGSs can also be used to assess propensity to a condition that may never be expressed phenotypically, highlighting shared genetic risk between traits and health conditions [8, 9]. Indeed, recent evidence showed that the polygenic underpinning of AD overlaps with general cognitive ability [10]. While steeper cognitive decline frequently heralds the onset of dementia spectrum [11], similar to dementia, individual differences in cognitive function are also driven by thousands of common genetic markers scattered across the whole genome [12, 13]. More recently, it was highlighted that polygenetic susceptibility to schizophrenia is associated with developmental cognitive deficit in the general population of adults [14]. The link between dementia and schizophrenia is further reiterated by findings demonstrating that schizophrenia is associated with a more than two-fold higher risk of all-cause dementia [15]. Thus, applying PGSs for these traits to estimating an individual risk for dementia diagnosis may provide insights into the genetic make-up of this complex disease informing the search for its biological mechanisms.

Nonetheless, lifestyle factors also contribute to individual-level risk of dementia. Accumulating evidence showed that dietary intake of fruit and vegetables, which serve as primary sources of antioxidants [16], is associated with dementia risk [16, 17]. However, this relationship of fruit and vegetables consumption with dementia risk is inconclusive [17] and susceptible to misinterpretation given previous studies have not accounted for genetic factors, which may also influence dietary habits [18, 19]. Indeed, the results from the twin and family studies alluded to a genetic effect on the number of calories consumed and on the preference for specific nutrients and food items [18].

Because genetic variants are determined randomly at conception and segregated independently of environmental influences, it is often assumed that a genetic susceptibility to dementia is deterministic. However, the genetic risk might be attenuated by a favourable dietary intake of fruit and vegetables [20]. Similarly, inherited DNA variation and dietary intake may contribute independently to a susceptibility to dementia. It is equally feasible that higher genetic propensity may exacerbate the effect of lower fruit and vegetable intake in moderating the risk of future dementia diagnosis. A clearer understanding of this gene-by-environment (G × E) interaction will help understand how genetic predisposition to dementia interacts with the daily intake of fruit and vegetables in determining risk of dementia diagnosis in the general population.

Therefore, we used a large population-representative cohort of older adults to investigate whether higher PGSs for AD, schizophrenia, and general cognition, chosen a priori to index genetic variants associated with ageing-related conditions [3, 10, 14], were associated with dementia diagnosis 10 years later. We further investigated the interactions between these PGSs with daily intake of fruit and vegetables in relation to the risk of dementia diagnosis. For prevention and intervention purposes, it is important to show if daily intake of fruit and vegetables precedes the risk for dementia onset. If it does, then interventions that successfully increase daily intake of fruit and vegetables may also translate into a reduced risk for dementia in the following years. Therefore, the fruit and vegetable daily intake was measured at baseline only. We hypothesised that there would be significant interaction effects between PGSs and the baseline daily intake of fruit and vegetables in association with the dementia diagnosis during the 10-year follow-up period.

## Methods and materials

### Sample

**Fig. 1:**
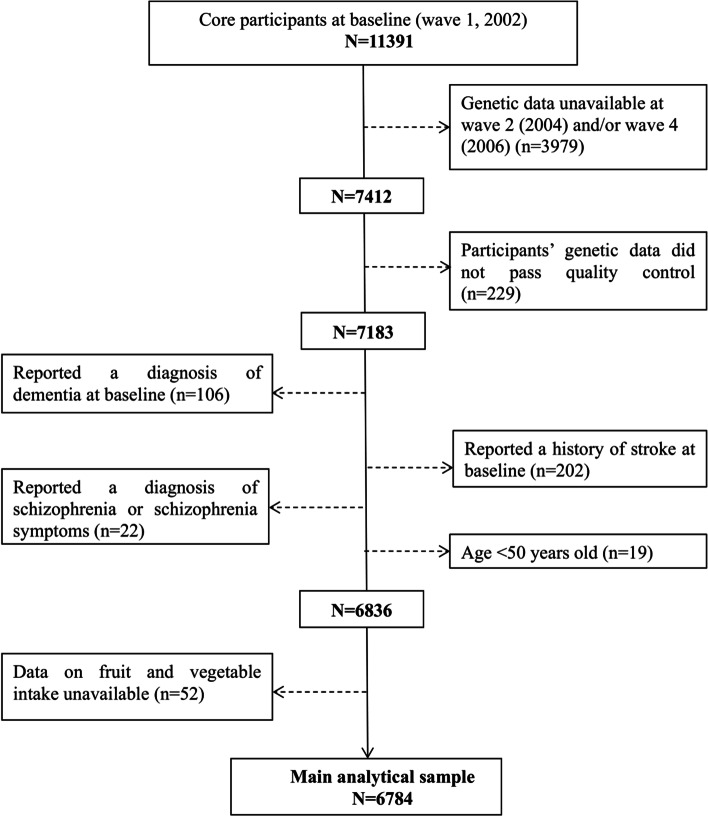
Flowchart depicting the selection process of the analytical sample

Data were drawn from the English Longitudinal Study of Ageing (ELSA) study, which is a nationally representative sample of the English population aged ≥50 years [21]. The ELSA study started in 2002–2003 (wave 1) with participants recruited from the Health Survey for England, who were then followed-up every 2 years. To ensure that individuals in their early fifties continued to be represented in the ELSA cohort, the sample is periodically refreshed with younger participants to ensure that the full age spectrum is maintained [21]. Comparisons of ELSA with the national census showed that the baseline sample was representative of the non-institutionalised general population aged 50 and above in the United Kingdom [21]. As participants were asked about their fruit and vegetable intake beginning at wave 3 (2006–2007) for the core members, or wave 4 (2008–2009) for those who joined the study through a refreshment sample [22], these waves formed our baseline. Follow-up data were taken from wave 5 (2010–2011) to wave 8 (2016–2017). We excluded participants with diagnosed dementia, stroke, and schizophrenia at baseline. The full process of sample selection is depicted in Fig. 1. The rates of attrition and mortality in our sample during a 10-year follow-up period are presented in Additional file 1: Table 1. Ethical approval for each of the ELSA waves was granted by the National Research Ethics Service (London Multicentre Research Ethics Committee). All participants gave informed consent. All methods and the study are GDPR compliant.

### Study variables

#### Ascertainment of dementia cases

Because there are gold standard indicators of dementia [23], dementia was ascertained at each wave using a physician made diagnosis of dementia or Alzheimer’s disease (AD). The diagnoses were reported at each wave by the participants who were capable of participating personally in the study, or by their carer. At each subsequent wave of follow-up, the participants who reported to have been given a diagnosis of dementia were asked if they still had the diagnosis, and reasons for disputing having the diagnosis of dementia. Only those participants who confirmed having dementia or AD diagnosis were classified as having dementia or AD. In the event that ELSA participants were unable to respond to the main interview themselves, the 16 items IQCODE was administered to an informant (family member or long-term caregiver), who knew the respondent very well. The purpose of the IQCODE was to compare the present functional and cognitive performance with the prior performance during the past 2 years employing a 5-point scale, each item is scored from 1 = much improved to 5 = much worse. A threshold of ≥3.38 or more on the IQCODE was used to define dementia [24, 25] with high-sensitivity (0.82) and specificity (0.84) [26]. In the articles that we have previously published using ELSA and cited here, we found that 3.38 performed well [3, 23, 27–30]. Overall, 83.5% of dementia cases were identified from reports of physician diagnosed dementia or AD, and 16.5% were identified based on the IQCODE score. This approach to identifying dementia incidence, including AD has been widely used in population-based cohorts reinforcing its validity [3, 28–30].

#### Survival age

Survival age was calculated from baseline age when all participants were dementia-free to the date when an ELSA participant received the first self-report physician diagnosis of dementia, or the diagnoses of dementia ascertained through the IQCODE assessment during follow-up. For those without dementia, the survival age was calculated using the period spanning from study entry until either the point of their death, the last wave before dropout, or wave 8. Mortality data were obtained from the National Health Service central register; all individuals included in the analyses provided written consent for the linkage.

#### Fruit and vegetable intake

Fruit intake was measured by asking participants how many small glasses of fruit juice and how many tablespoons of various types of fruits they consumed the previous day. Vegetable intake was assessed by asking participants how much salad was eaten using a cereal bowl as the standard and how many tablespoons of either vegetables or pulses were consumed the previous day. Consistent with recommendations from the World Health Organization [31] and the U.K. National Health Service which recommend consuming five or more servings of fruits and vegetables per day [32], the total number of fruit and vegetable eaten during 1 day was categorised into a binary variable measuring whether participants consumed the recommended a minimum of 5 daily servings of fruit and vegetables a day (< 5 vs. ≥5 portions) to lower risk of serious long-term conditions [22]. As such, our findings would allow for comparison with previous studies that have used this cut-off point when analysing fruit and vegetable consumption [22].

#### Covariates

The set of covariates included sex (male) and genetic ancestry (as was measured with principal components (see below)), was included among the covariates to account for any ancestry differences in genetic structures that could bias our results. Specifically, because the ELSA participants had a homogeneous European background, we did not expect population stratification to have a strong influence on our results; therefore, we included four principal components as covariates. Because the apolipoprotein E gene (*APOE*-ε4) is one of the most significant determinant of dementia risk [33, 34], the APOE-locus should be treated as an independent factor in analyses of dementia sample when calculating polygenic scores [34]. Therefore, we included the ε4 allele of the apolipoprotein E gene (*APOE*-ε4) status as a confounder in our analyses. Consistent with previous research [35], *APOE-ε4* status was defined according to absence (*APOE* ε2/2, ε2/3 and ε3/3) or presence (*APOE* ε2/4, ε3/4 and ε4/4) of *APOE*-ε4 alleles.

### Genetic data

#### Quality control

The genome-wide genotyping was performed at University College London Genomics in 2013–2014 using the Illumina HumanOmni2.5 BeadChips (HumanOmni2.5-4v1, HumanOmni2.5-8v1.3). Quality control followed specifications outlined in Additional file 1. To improve genome coverage, we imputed untyped quality-controlled genotypes to the Haplotype Reference Consortium [36, 37] using the University of Michigan Imputation Server [36]. Post-imputation, we kept variants that were genotyped or imputed at INFO> 0.95, in low linkage disequilibrium (*R*^2^ < 0.1) and with Hardy-Weinberg Equilibrium *p*-value> 10^− 5^. After the sample quality control 7,179,780 variants were retained for further analyses. An overview of the summary of these quality control steps are presented in Additional file 1: Table 2. To account for any ancestry differences in genetic structures that could bias results, principal components analysis was conducted retaining top principal components [38].

#### Polygenic scores

Polygenic score for AD (AD-PGS), schizophrenia (SZ-PGS) and general cognition (GC-PGS) were calculated using summary statistics from genome-wide association studies [39–41]. As creating polygenic scores based on pruning and *p*-value threshold has been criticised for discarding potentially important information and limiting prediction accuracy [42, 43], we calculated PGSs using polygenic risk score approach with continuous shrinkage (PRS-CS) [42]. PRS-CS utilises a Bayesian regression framework and places continuous shrinkage priors to address linkage disequilibrium without losing potentially important data. Simulated and real data analyses showed that PRS-CS improves the predictive performance of PGS over existing methods, such as LDpred [43], across a wide range of genetic architectures [42]. There were weak negative correlations between GC-PGS vs AD-PGS (*r*^2^ = − 0.04, *p* = 0.001), and GC-PGS vs SZ-PGS (*r*^2^ = − 0.16, *p* < 0.001) (Additional file 1: Table 5). To aid interpretability of the results, all PGSs were standardised (mean = 0, SD = 1). In this context, a 1 standard deviation increase in the explanatory variable is equivalent to a unit increase in the standardized version of the variable.

### Statistical analysis

#### Imputing missing values

In the present study, some variables had missing values (Additional file 1: Table 3). Given analyses of complete cases (i.e., subset with no missing data in any of variables included for analysis) can result in reduced power and precision of estimates [44, 45], we imputed missing values employing *missForest* in RStudio version 3.6.2 [46]. MissForest is a nonparametric imputation method based on random forest, which handles continuous and categorical variables equally well and accommodates non-linear relation structures [46, 47]. *MissForest* has been shown to outperform the well-known imputation methods, such as *k*-nearest neighbours and parametric multivariate imputation by chained equations (MICE) in the presence of large proportion of missingness, non-linearity and variable interactions. To improve the quality of the imputed missing values we additionally included auxiliary variables [48], such as baseline age, marital status, smoking and presence of any lifelong health conditions. Distributions of the variables before and after imputation are presented in Additional file 1: Table 4, which demonstrate that imputed values are closely aligned with the observed values for all imputed variables.

#### Association analyses

To investigate the impact of AD-PGS, SZ-PGS and GC-PGS and the baseline daily intake of fruit and vegetables on the risk of dementia diagnosis during the 10-year follow-up period, we utilised the accelerated failure time (AFT) survival model for right-censored data separately for each PGS. To identify the best-fitting parametric model (i.e., exponential, Weibull, lognormal and generalized gamma), we employed the Akaike information criterion [49, 50], which showed that the Weibull model was the most appropriate for our analyses. For each PGS, we constructed the hazard function based on the survival age, as this approach is not restricted to the length of the follow-up period. To investigate whether our findings were applicable to all dementia or were specific to AD cases, we repeated the analyses limiting them to either AD cases only or removing individuals with a diagnosis of AD from the sample (non-AD cases).

#### Interactions

Interactions between PGSs and the baseline daily fruit and vegetable intake were investigated using multiplicative and additive models. The multiplicative model tests interaction as the departure from multiplicativity according to which the combined effect of two risk factors differs from the product of their individual effects; whereas, the additive interaction tests whether the combined effect of two risk factors differs from the sum of their individual effects [51]. To present the results from the additive interactions, we derived the relative excess risk due to interaction (RERI) and attributable proportion due to interaction (AP) [51, 52], with corresponding 95% confidence intervals. Here, RERI = 0; AP = 0 shows no interaction or exactly equal to additivity of the individual effects of the two risk factors; RERI> 0; AP > 0 highlight positive interaction or more than additivity of the individual effects of the two risk factors; RERI< 0; AP < 0 show negative interaction or less than additivity of the individual effects of the two risk factors.

#### Sensitivity analyses

We re-ran AFT survival models using the total number of fruit and vegetable eaten as a continuous variable (Additional file 1: Tables 6–7). As the frequency distribution of the total number of fruit and vegetable eaten was severely skewed, this variable was normalised by taking the logarithm to base 10 (log10_fruits & vegetables_) to allow the use of parametric models (Additional file 1: Figure 1). The *p* < 0.05 was considered statistically significant. Association analyses were conducted in STATA release 14 (STATA CorpLP, USA).

## Results

### Sample characteristics

The sample comprised 6784 individuals for whom the quality-controlled genome-wide genotyping and dementia status during follow-up were available (Table 1). Of these, 271 (4.0%) were classified as having dementia (i.e., cases) over the 10-year follow-up, and 6513 (96.0%) remained dementia-free (i.e., controls). Of all dementia cases, 69 (25.5%) had the diagnosis of AD and 109 (40.2%) were APOE-ε4 carriers. The baseline mean age for the entire sample was 64.5 years (standard deviation (SD) = 9.3, median = 63, range = 50–101). 64.6% (*N* = 175) of future dementia cases compared to 53.0% (*N* = 3452) controls consumed < 5 portions of fruit and vegetables a day at baseline (*x*^2^ = 14.0, *p* < 0.001).

**Table 1:** Baseline sample characteristics of ELSA participants

	Total sample ***N*** = 6784	Dementia cases ***N*** = 271 (4.0%)	Controls ***N*** = 6513 (96.0%)	Test statistics		
	***N*** (%) / Mean (SD)	***N*** (%) / Mean (SD)	***N*** (%) / Mean (SD)	***t***/***x***^**2**^	DF	***p***-value
*Baseline sample characteristics*						
Age at baseline (years)	64.5 (9.3)	73.2 (8.6)	64.2 (9.2)	−15.95	6782	<.001
Men	3135 (46.2)	117 (43.2)	3018 (46.3)	1.05	1	.31
*APOE-ε4* present	1710 (25.2)	109 (40.2)	1601 (24.6)	33.76	1	<.001
Not married	4685 (69.1)	162 (59.8)	4523 (69.5)	11.38	1	.001
Currently a smoker	1068 (15.7)	40 (14.8)	1028 (15.8)	0.21	1	.65
Presence of any lifelong limiting conditions	2114 (31.2)	119 (43.9)	1995 (30.6)	21.39	1	<.001
< 5 portions of fruit and vegetable intake daily	3627 (53.5)	175 (64.6)	3452 (53.0)	14.01	1	<.001

Abbreviations: *AD* Alzheimer’s disease; *APOE-ε4* two ε4 alleles of the Apolipoprotein E gene; *SD* standard deviation; *DF* degrees of freedom

### PGS, fruit and vegetable intake

Independently from PGSs, consuming < 5 portions of fruit and vegetables a day was associated with a higher risk for dementia diagnosis ranging from 33 to 37% (Table 2). One standard deviation (1-SD) increase in AD-PGS was associated with a 24% greater risk of dementia diagnosis (HR = 1.24, 95%CI = 1.01–1.51) and a 47% greater risk of AD (HR = 1.47, 95%CI = 1.00–2.18) during the 10-year follow-up. There was a significant multiplicative interaction effect between SZ-PGS and the baseline daily intake of fruit and vegetables in relation to AD diagnosis risk during follow-up (HR = 1.66, 95%CI = 1.05–2.64). Accordingly, 1-SD increase in SZ-PGS was associated with an increased risk of AD diagnosis by an average 66% in participants who consumed < 5 portions of fruit or vegetables a day at baseline. Having a higher PGS for general cognition was associated with a reduced risk for non-AD diagnosis (HR = 0.80, 95%CI = 0.65–0.98). There was a significant additive interaction between GC-PGS and < 5 portions of the baseline daily intake of fruit and vegetables in association with AD diagnosis during the 10-year follow-up (RERI = 0.70, 95%CI = 0.09–4.82; AP = 0.36, 95%CI = 0.17–0.66) (Additional file 1: Table 6).

**Table 2:** Multivariate AFT model estimating associations of PGSs for ageing-related traits and risk for dementia diagnosis

	Total sample	Alzheimer’s diagnosis	non-AD cases
	HR (95% CI)	HR (95% CI)	HR (95% CI)
***AD-PGS***			
PGS	1.24 (1.01–1.51) *	1.47 (1.00–2.18) *	1.16 (0.92–1.47)
< 5 portions of fruit and vegetables	1.33 (1.03–1.73) *	1.48 (0.85–2.56)	1.30 (0.97–1.75)
PGS × < 5 portions fruits & vegetables	1.05 (0.83–1.32)	0.91 (0.58–1.44)	1.10 (0.84–1.44)
***SZ-PGS***			
PGS	0.89 (0.74–1.07)	0.83 (0.58–1.18)	1.23 (0.97–1.55)
< 5 portions of fruit and vegetables	1.37 (1.07–1.76) **	1.41 (0.85–2.36)	1.33 (0.99–1.78)
PGS × < 5 portions fruits & vegetables	0.97 0.77–1.23)	1.66 (1.05–2.64) *	0.97 (0.73–1.30)
***GC-PGS***			
PGS	1.10 (0.90–1.34)	1.30 (0.88–1.92)	0.80 (0.65–0.98) *
< 5 portions of fruit and vegetables	1.34 (1.04–1.72) *	1.46 (0.87–2.44)	1.37 (1.02–1.84) *
PGS × < 5 portions fruits & vegetables	1.12 (0.88–1.44)	0.78 (0.48–1.29)	1.03 (0.78–1.35)

Abbreviations: *AFT* Accelerated Failure Time; *PGS* polygenic score; *AD-PGS* polygenic score for Alzheimer’s disease; *SZ-PGS* polygenic score for schizophrenia; *GC-PGS* polygenic score for general cognition; *HR* hazard ratio; *CI* confidence interval; *APOE-ε4* the ε4 allele of the apolipoprotein E gene

× represents an interaction between the two factors; interactions are presented based on multiplicative interaction model

* *p*-value ≤ .05; ** *p*-value ≤ .01; *** *p*-value ≤ .001

### Sensitivity analyses

When using a continuous variable of the baseline daily number of fruit and vegetable consumed, the results related to AD-PGS remained unchanged (Additional file 1: Table 6). 1-SD increase in SZ-PGS was associated with a greater risk of dementia diagnosis by an average 24% during the 10-year follow-up (HR = 1.24, 95%CI = 1.04–1.49). There was a significant multiplicative interaction effect between SZ-PGS and the baseline daily intake of fruit and vegetables in relation to the risk of dementia (HR = 0.84, 95%CI = 0.71–0.99) and AD diagnosis (HR = 0.64, 95%CI = 0.47–0.88) during follow-up. Accordingly, 1-SD increase in SZ-PGS was associated with a decreased risk of dementia and AD diagnosis by an average 16 and 36%, respectively, per every portion of fruit or vegetables. All previous findings related to GC-PGS and participants consuming > 5 servings of fruit and vegetables did not remain when we used the baseline fruit and vegetable consumption as a continuous variable (Additional file 1: Tables 6–7).

## Discussion

To our knowledge, this is the first study to have investigated interactions between polygenic propensity for AD, schizophrenia and general cognition, and the baseline daily intake of fruit and vegetables in estimating risk for dementia and AD diagnoses in the following 10 years using a large population-representative sample of older adults. Consistent with the genetic liability threshold model according to which the combined effect of many genetic risk variants with other factors causes an individual to cross the threshold leading to the development of a condition [53], we identified significant interactions between the polygenic propensity for schizophrenia and the baseline daily fruit and vegetable intake in association with an increased risk for dementia and AD onset in the following 10 years. This, combined with an assertion that a long prodromal phase of AD allows for the modification of a lifestyle-related factor toward reducing AD risk [54], may suggest that the earlier a healthy lifestyle approach is adopted the more protective against AD it would be.

Consistent with the evidence-based support for fruit and vegetable intake as an important factor influencing risk for dementia [16, 17], we found that consuming less than 5 servings of fruit and vegetable a day was associated with an increased risk of dementia in the following 10 years independently from the genetic propensity for this disease and the APOE-ε4 status. As pathologic processes involved in dementia include oxidative stress [55] and inflammation [56], consuming less fruit and vegetable than recommended to lower risk of serious long-term conditions [22] may lead to diminished numbers of antioxidant and anti-inflammatory compounds present in the brain to decrease risk for oxidative damage, down-regulate inflammation and strengthen the neurons’ antioxidant defence [57]. Consequently, the brain’s ability to withstand more pathology before clinical symptoms of dementia become detectable may be significantly decreased. While these findings reiterate that increasing intake of fruits and vegetables to at least to 5 portions a day may lower the risk of dementia; the non-significant relationship of fruit and vegetable intake with AD risk may reflect the relatively small sample of AD cases available in the ELSA cohort.

Our results further indicate that a higher aggregate of loci for AD may exert its effect by accelerating the clinical presentation of dementia, and to a greater extent of AD. This finding reiterates the results from previous studies [3, 6, 7] and highlights the existence of specific population subgroups that may be at higher risk of dementia based on their polygenetic loading. Some of the mechanisms put forward to explain this relationship assert that common genetic variants associated with AD may affect the immune response, regulation of endocytosis, cholesterol transport and protein ubiquitination [58]. Moreover, we found that higher GC-PGS, which services as an indicator for polygenic predisposition for higher general cognition [12], was associated with a reduced risk for non-AD diagnosis during the 10-year follow-up period in participants. This is consistent with the notion that a higher polygenic propensity to general cognition serves as a protective factor against dementia-related outcomes. However, the interaction effect showed that the protective effect of higher polygenic propensity for a better cognition may be insufficient to counteract the impact of consuming less fruits and vegetables a day than recommended by the World Health Organization [31] and the U.K. National Health Service against developing the non-AD in the following 10 years. Nonetheless, these results did not remain when the analyses were repeated using the baseline fruit and vegetable consumption as a continuous variable. This may imply that categorising the total consumption of fruit and vegetable based on the recommended a minimum of 5 daily servings of fruit and vegetables a day (< 5 vs. ≥5 portions) to lower risk of serious long-term conditions [22] captures important risks that the continuous variable does not.

We further detected a modifying effect of polygenic predisposition to schizophrenia on the association between consuming less than 5 portions of fruit and vegetable and AD diagnosis in the following 10 years. Specifically, a higher genetic load of schizophrenia alleles appears to increase risk of AD diagnosis, but not dementia, among older individuals who did not have a diet rich in fruits and vegetables. These findings, in combination with previous reports that AD onset is often associated with psychosis [59–62], may suggest that multiple differently regulated aetiological pathways may give rise to similar clinical presentation, but only in the context of environmental exposures, such as daily intake of fruit and vegetables. As neuroinflammation has been implicated in both of AD and schizophrenia [63, 64], it may be hypothesised that fruit and vegetables attenuate the role played by genetic markers for schizophrenia in association with AD diagnosis via antioxidant and anti-inflammatory properties [16].

### Methodological considerations

In the present study, we analysed a large sample of adults who were nationally representative of older adults in England. Our study further included an almost equal proportion of women and men all of whom were from socio-economically diverse backgrounds. The present study has a unique value given the current sparseness of data on diet before dementia onset [19]. The further strengths of this study included its prospective collection of information on dementia diagnose and risk factors. Nonetheless, several limitations warrant a discussion. Using doctor diagnosis to identify most dementia cases may imply that the presented dementia and AD cases might have been underestimated [3]. Although dementia and AD were ascertained using a combined algorithm based on a physician made diagnosis and a higher score on the informant reports (IQCODE), it is still reliant on a self-reported diagnosis reported by either the participant themselves or their carers and render more severe cases. As it is estimated that dementia remains undetected in almost 30 to 50% of primary care patients in the UK [65], we cannot exclude a possibility that some participants within the “dementia-free’ group may have been the preclinical stages of dementia and who, if followed for long enough, might eventually develop dementia. Because all covariates that were included in the models were set at birth, we minimised chances of collider bias effecting our findings [66]; on the other hand, however, because of this nature of the analyses, we did not adjust the confounding effect on the dietary intake.

It is being argued that when investigating the G × E interaction to properly control for confounders, the covariate × environment and the covariate × gene interaction terms in the same model ought to be included [67]. However, considering that we have 13 covariates and small numbers in the outcomes, entering 36 interaction terms in each model is likely to lead to overfitting [68]. Therefore, to avoid overfitting, we were unable to adjust our models for interactions between the covariates as advised [67]. Because the construction of PGSs is largely dependent on the availability of the summary statistics from genome-wide association studies (GWASs), the vast majority of which is based on the participants of European descent [69], further work is necessary to develop the PGSs model in non-white populations. Further, the methodology employed to collect data on the baseline daily consumption of fruit and vegetables may not necessarily represent a long-term habit. The measures of the baseline fruit and vegetable consumption may have been restricted because intake during the previous day rather than a typical day was queried [22]. The reason for focusing on the previous day is that memory is stronger for recent experiences. Finally, the proportion of missing data in the present study was comparable to other population-representative longitudinal cohort of similar design [21, 70, 71] and within the range for missForest to handle it efficiently [72, 73].

## Conclusion

Our findings reiterate that increasing intake of fruits and vegetables to at least 5 portions a day may lower the risk of dementia and AD independently from the genetic propensity for this disease and the APOE-ε4 status. Consuming at least 5 portions of fruit and vegetable a day may lower the risk of dementia and AD, especially among those who have higher polygenic predisposition to AD and schizophrenia. Future emphasis on promoting healthy behaviours especially in later life, although challenging, may prove valuable in reducing the public health burden of dementia.

## Supplementary Information

**Additional file 1.**

## Data Availability

The English Longitudinal Study of Ageing (ELSA) was developed by a team of researchers based at University College London, the Institute for Fiscal Studies and the National Centre for Social Research. The data are linked to the UK Data Archive and freely available through the UK data services and can be accessed here: https://discover.ukdataservice.ac.uk.

## Abbreviations

AD: Alzheimer’s diagnosis
APOE: Apolipoprotein E
ELSA: English Longitudinal Study of Ageing
GC: General cognition
GWAS: Genome-wide association study
IQCODE: Informant Questionnaire on Cognitive Decline in the Elderly
PGS: Polygenic score
PRS-CS: Polygenic risk score approach with continuous shrinkage
RERI: Relative excess risk due to interaction
SZ: Schizophrenia
