# Supplementary material for: Interplay between polygenic propensity for ageing-related traits and the consumption of fruits and vegetables on future dementia diagnosis
Source: BMC Psychiatry. 2022 Jan 30;22:75. doi: 10.1186/s12888-022-03717-5 (PMC8801085; doi:10.1186/s12888-022-03717-5)
Supplement: Supplementary file 1 — Additional file 1. [file 12888_2022_3717_MOESM1_ESM.docx]

**ADDITIONAL FILE 1**

**Table 1. presents rates of attrition and mortality in adults aged ≥50 years from the ELSA study during a 10-year follow-up period**

|  | Baseline  N=6784  (2006-2009) | Wave 5  (2010-2011) | Wave 6  (2012-2013) | Wave 7  (2014-2015) | Wave 8  (2016-2017) |
| --- | --- | --- | --- | --- | --- |
|  |  | N (%) | N (%) | N (%) | N (%) |
| Followed-up | - | 5274 (77.7) | 4939 (72.8) | 4413 (65.1) | 3851 (56.7) |
| Developed dementia | - | 89 (1.7) | 141 (2.8) | 201 (4.5) | 271 (7.0) |
| Lost to follow-up | - | 1522 (23.3) | 1888 (27.8) | 2455 (36.2) | 2933 (43.3) |
| Died | - | 731 (10.8) | 949 (14.0) | 1212 (17.9) | 1457 (21.5) |

**Table 2. An overview of the summary of full QC procedure employed in the ELSA study and how many variants and/or participants were lost at each step.**

| Quality Control steps in ELSA | | | |
| --- | --- | --- | --- |
| *Lost due to SNP-based QC* | | *n* | % |
|  | Missing SNPs (0.02) | 41614 | 1.87 |
|  | Autosomal SNPs | 48578 | 2.18 |
|  | MAF 0.01 | 759972 | 34.07 |
|  | Update rsids | 2284 | 0.10 |
|  | HWE (0.0001) | 6079 | 0.27 |
|  |  |  |  |
|  | *Total removed* | *858527* | *38.49* |
|  | *Total remaining* | *1372240* | *61.51* |
| *Lost due to Individual-based QC* | |  |  |
|  | Missingness (0.02) | 39 | 0.53 |
|  | Heterogeneity | 76 | 1.03 |
|  | Sex discordance | 5 | 0.07 |
|  | Ancestry outliers | 64 | 0.86 |
|  | Relatedness/Duplicates | 5 | 0.07 |
|  | Unique IDs are not present | 41 | 0.50 |
|  |  |  |  |
|  | *Total removed* | *229* | *3.09* |
|  | *Total remaining* | *7183* | *96.91* |

HWE, Hardy-Weinberg equilibrium; MAF, minor allele frequency; SNP, single nucleotide polymorphisms

**Table 3. Distribution of missing and observed variables at baseline and follow-up in ELSA**

| **Variables at baseline** | | **N observed** | **% observed** | **N missing** | **% missing** |
| --- | --- | --- | --- | --- | --- |
|  | |  |  |  |  |
|  | Age (years) | 6784 | 100 | 0 | 0 |
|  | Male gender | 6784 | 100 | 0 | 0 |
|  | *APOE-ε4* present | 6784 | 100 | 0 | 0 |
|  | Not married | 6784 | 100 | 0 | 0 |
|  | Currently a smoker | 6390 | 94.19 | 394 | 5.81 |
|  | Presence of any lifelong limiting conditions | 6783 | 99.99 | 1 | 0.01 |
|  | Fruit and vegetable daily intake | 3966 | 58.46 | 2817 | 41.54 |
|  | AD | 6580 | 96.99 | 204 | 3.01 |
|  | Dementia all cases | 6784 | 100 | 0 | 0 |

Abbreviations: AD, Alzheimer’s disease; APOE-ε4, two ε4 alleles of the Apolipoprotein E gene.

**Table 4. Distribution of the variables before and after imputation**

| **Variables** | | **Variables before imputation**  ***N*=6784** | **Variables after imputation**  ***N*=6784** |
| --- | --- | --- | --- |
|  |  | **N (%) / mean (SD)** | **N (%) / mean (SD)** |
|  | Age (years) | 64.53 (9.3) | 64.53 (9.3) |
|  | Male gender | 3135 (46.2) | 3135 (46.2) |
|  | *APOE-ε4* present | 1710 (25.2) | 1710 (25.2) |
|  | Not married | 4685 (69.1) | 4685 (69.1) |
|  | Currently a smoker | 971 (14.3) | 1068 (15.7) |
|  | Presence of any lifelong limiting conditions | 2115 (31.2) | 2114 (31.2) |
|  | Fruit and vegetable daily intake | 3493 (51.5) | 3452 (53.0) |
|  | Alzheimer’s disease (AD) cases | 67 (1.00) | 69 (1.0) |
|  | Dementia all cases | 271 (4.0) | 271 (4.0) |

Abbreviations: APOE-ε4, two ε4 alleles of the Apolipoprotein E gene; SD, standard deviation

**Table 5**. Correlations between polygenic scores

|  |  | AD-PGS | | SZ-PGS | | GC-PGS | |
| --- | --- | --- | --- | --- | --- | --- | --- |
|  |  | Corelation | P-value | Corelation | P-value | Corelation | P-value |
| AD-PGS |  | 1.0000 | - |  |  |  |  |
| SZ-PGS |  | -0.0052 | 0.6699 | 1.0000 | - |  |  |
| GC-PGS |  | -0.0397 | 0.0011 | -0.1583 | <0.001 | 1.0000 | - |

Abbreviations: PGS, polygenic score; AD-PGS, polygenic score for Alzheimer’s disease; SZ-PGS, polygenic score for schizophrenia; GC-PGS, polygenic score for general cognition

**Table 6. Present additive interactions from Multivariate Accelerated Failure Time model, which estimated associations of PGSs for age-related traits and risk for dementia diagnosis in older adults during the 10-year follow-up; in these analyses the total number of fruit and vegetable eaten during 1 day was categorised into a binary variable measuring whether participants consumed the recommended a minimum of 5 daily servings of fruit and vegetables a day (<5 *vs*. ≥5 portions) to lower risk of serious long-term conditions**

| PGSs and interactions with <5 portions fruits & vegetables | |  | **Total sample** |  | **Alzheimer’s diagnosis** |  | **non-AD cases** |
| --- | --- | --- | --- | --- | --- | --- | --- |
|  |  |  | **RERI (95% CI) /**  **AP (95% CI)** |  | **RERI (95% CI) /**  **AP (95% CI)** |  | **RERI (95% CI) /**  **AP (95% CI)** |
| *AD-PGS* | |  |  |  |  |  |  |
|  | PGS |  | - |  | - |  | - |
|  | <5 portions of fruit and vegetables |  | - |  | - |  | - |
|  | PGS × <5 portions fruits & vegetables |  | 0.16 (-0.18 to 0.41) /  0.09 (-0.20 to 0.35) |  | 0.04 (-0.36 to 4.30) /  0.02 (-0.72 to 0.53) |  | 0.20 (-0.14 to 1.49) /  0.12 (-0.19 to 0.40) |
|  |  |  |  |  |  |  |  |
| *SZ-PGS* | |  |  |  |  |  |  |
|  | PGS |  | - |  | - |  | - |
|  | <5 portions of fruit and vegetables |  | - |  | - |  | - |
|  | PGS × <5 portions fruits & vegetables |  | -0.07 (-0.20 to 0.50) /  -0.06 (-0.33 to 0.21) |  | -0.27 (-0.39 to 2.68) /  -0.18 (-1.05 to 0.44) |  | -0.04 (-0.15 to 0.61) /  -0.04 (-0.29 to 0.25) |
|  |  |  |  |  |  |  |  |
| *GC-PGS* | |  |  |  |  |  |  |
|  | PGS |  | - |  | - |  | - |
|  | <5 portions of fruit and vegetables |  | - |  | - |  | - |
|  | PGS × <5 portions fruits & vegetables |  | 0.21 (-0.12 to 1.24) /  0.13 (-0.14 to 0.38) |  | 0.70 (0.09 to 4.82) /  0.36 (0.17 to 0.66) |  | 0.03 (-0.26 to 1.25) /  0.02 (-0.37 to 0.35) |

Abbreviations: PGS, polygenic score; AD-PGS, polygenic score for Alzheimer’s disease; SZ-PGS, polygenic score for schizophrenia; GC-PGS, polygenic score for general cognition; HR, hazard ratio; CI, confidence interval; APOE-ε4, the ε4 allele of the apolipoprotein E gene; RERI, relative excess risk due to interaction; AP, attributable proportion; RERI=0; AP=0: no interaction or exactly equal to additivity of the individual effects of the two risk factors; RERI>0; AP>0: positive interaction or more than additivity of the individual effects of the two risk factors; RERI<0; AP<0: negative interaction or less than additivity of the individual effects of the two risk factors.

× represents an interaction between the two factors; interactions are presented based on multiplicative interaction model

**Table 7. Multivariate AFT model estimating associations of PGSs for age-related traits and risk for dementia diagnosis**

| **PGSs and interactions with <5 portions fruits & vegetables** | |  | **Total sample** |  | **Alzheimer’s diagnosis** |  | **non-AD cases** |
| --- | --- | --- | --- | --- | --- | --- | --- |
|  |  |  | **HR (95% CI)** |  | **HR (95% CI)** |  | **HR (95% CI)** |
| ***AD-PGS*** | |  |  |  |  |  |  |
|  | PGS |  | 1.25 (1.02-1.54) * |  | 1.47 (1.00-2.14) * |  | 1.17 (0.92-1.49) |
|  | Portions of fruit and vegetables |  | 1.12 (0.92 - 1.36) |  | 1.11 (0.75 - 1.63) |  | 1.12 (0.90-1.42) |
|  | PGS × portions fruits & vegetables |  | 0.98 (0.84 - 1.15) |  | 0.81 (0.61 - 1.09) |  | 1.06 (0.88-1.26) |
|  |  |  |  |  |  |  |  |
| ***SZ-PGS*** | |  |  |  |  |  |  |
|  | PGS |  | 1.24 (1.04 - 1.49) ** |  | 1.31 (0.93 - 1.862) |  | 1.23 (0.97-1.55) |
|  | Portions of fruit and vegetables |  | 1.11 (0.92 - 1.34) |  | 1.01 (0.71 1.44) |  | 1.33 (0.99-1.78) |
|  | PGS × portions fruits & vegetables |  | 0.84 (0.71 - 0.99) * |  | 0.64 (0.47 - 0.88) ** |  | 0.97 (0.73-1.30) |
|  |  |  |  |  |  |  |  |
| ***GC-PGS*** | |  |  |  |  |  |  |
|  | PGS |  | 0.92 (0.77 - 1.10) |  | 1.07 (0.76 - 1.52) |  | 1.21 (0.98 - 1.50) |
|  | Portions of fruit and vegetables |  | 1.11 (0.92 - 1.33) |  | 0.98 (0.69 - 1.41) |  | 1.15 (0.92 - 1.43) |
|  | PGS × portions fruits & vegetables |  | 1.04 (0.89 - 1.22) |  | 1.12 (0.82 - 1.52) |  | 0.93 (0.77 - 1.12) |

Abbreviations: AFT, Accelerated Failure Time; PGS, polygenic score; AD-PGS, polygenic score for Alzheimer’s disease; SZ-PGS, polygenic score for schizophrenia; GC-PGS, polygenic score for general cognition; HR, hazard ratio; CI, confidence interval; APOE-ε4, the ε4 allele of the apolipoprotein E gene

× represents an interaction between the two factors; interactions are presented based on multiplicative interaction model

* *p*-value ≤ .05; ** *p*-value ≤ .01; *** *p*-value ≤ .001

**Table 8. Multivariate AFT model estimating associations of PGSs for age-related traits and risk for dementia diagnosis; in these analyses the total number of fruit and vegetable eaten was used as a continuous variable.**

| **PGSs and interactions with portions fruits & vegetables** | |  | **Total sample** |  | **Alzheimer’s diagnosis** |  | **non-AD cases** |
| --- | --- | --- | --- | --- | --- | --- | --- |
|  |  |  | **RERI (95% CI) /**  **AP (95% CI)** |  | **RERI (95% CI) /**  **AP (95% CI)** |  | **RERI (95% CI) /**  **AP (95% CI)** |
| ***AD-PGS*** | |  |  |  |  |  |  |
|  | PGS |  | - |  | - |  | - |
|  | Portions of fruit and vegetables |  | - |  | - |  | - |
|  | PGS × portions fruits & vegetables |  | 0.01(-0.15 to 0.50) /  0.004 (-0.19 to 0.21) |  | -0.25 (-0.29 to 1.05) /  -0.19 (-0.64 to 0.27) |  | 0.10 (-0.09 to 0.76) /  0.07 (-0.21 to 0.29) |
|  |  |  |  |  |  |  |  |
| ***SZ-PGS*** | |  |  |  |  |  |  |
|  | PGS |  | - |  | - |  | - |
|  | Portions of fruit and vegetables |  | - |  | - |  | - |
|  | PGS × portions fruits & vegetables |  | 0.04 (-0.06 to 0.36) /  0.04 (-0.09 to 0.20) |  | 0.12 (-0.02 to 1.32) /  0.10 (-0.04 to 0.41) |  | 0.01 (-0.07 to 0.39) /  0.01 (-0.13 to 0.21) |
|  |  |  |  |  |  |  |  |
| ***GC-PGS*** | |  |  |  |  |  |  |
|  | PGS |  | - |  | - |  | - |
|  | Portions of fruit and vegetables |  | - |  | - |  | - |
|  | PGS × portions fruits & vegetables |  | -0.19 (-0.28 to 0.16) /  -0.16 (-0.40 to 0.08) |  | -0.47 (-0.32 to 0.06) /  -0.54 (-1.04 to 0.02) |  | -0.07 (-0.21 to 0.48) /  -0.05 (-0.30 to 0.20) |

Abbreviations: PGS, polygenic score; AD-PGS, polygenic score for Alzheimer’s disease; SZ-PGS, polygenic score for schizophrenia; GC-PGS, polygenic score for general cognition; HR, hazard ratio; CI, confidence interval; APOE-ε4, the ε4 allele of the apolipoprotein E gene; RERI, relative excess risk due to interaction; AP, attributable proportion; RERI=0; AP=0: no interaction or exactly equal to additivity of the individual effects of the two risk factors; RERI>0; AP>0: positive interaction or more than additivity of the individual effects of the two risk factors; RERI<0; AP<0: negative interaction or less than additivity of the individual effects of the two risk factors.

× represents an interaction between the two factors; interactions are presented based on multiplicative interaction model

**Figure 1. Distribution of portions fruits & vegetables post log-transformation**

**
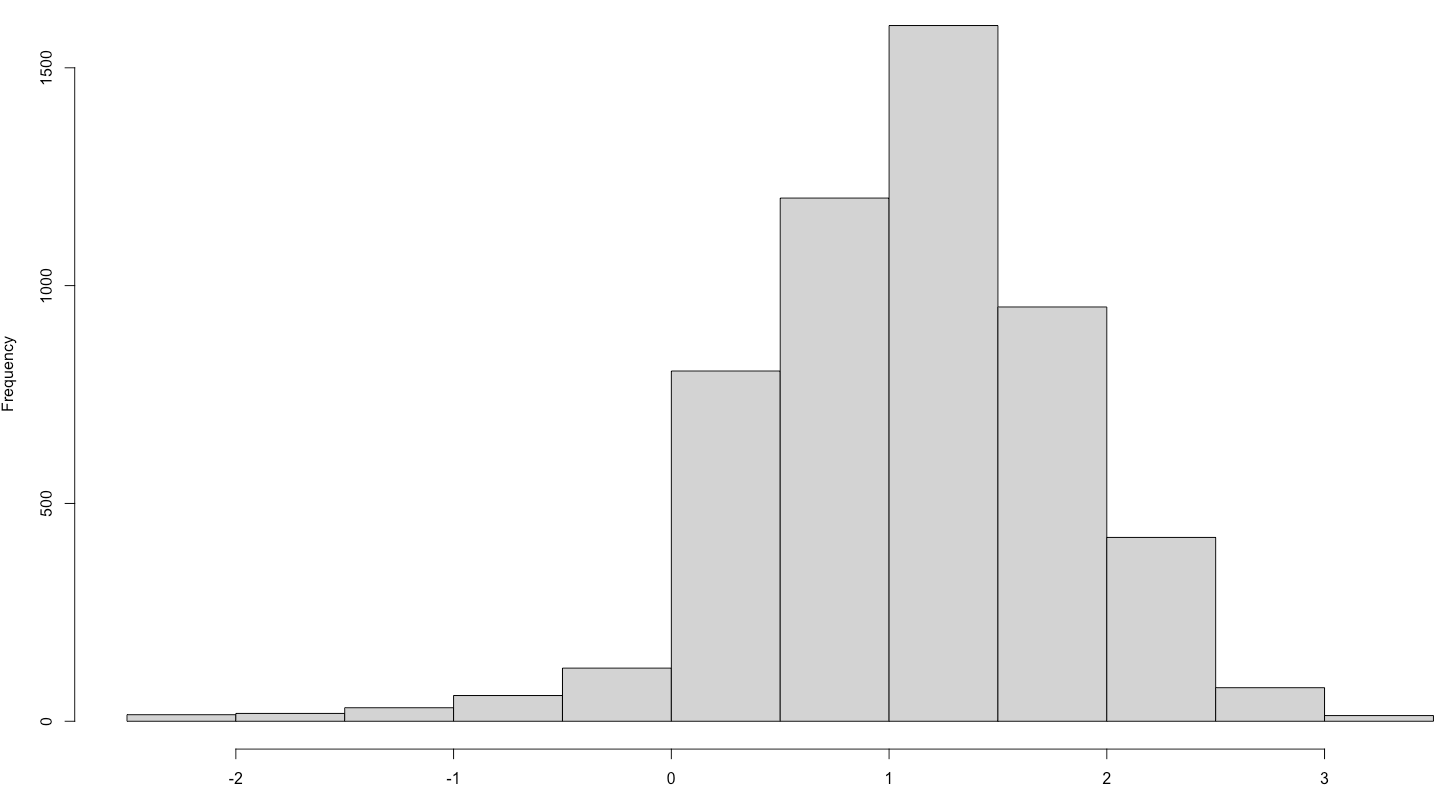
**
